# Low cross reactivity between wild type and deamidated AAV can lead to false negative results in immune monitoring T-cell assays

**DOI:** 10.3389/fimmu.2023.1211529

**Published:** 2023-07-04

**Authors:** So Jin Bing, Stephanee Warrington, Ronit Mazor

**Affiliations:** Division of Gene Therapies II, Office of Gene Therapy CMC, Center for Biologics Evaluation and Research, U. S. Food and Drug Administration, Silver Spring, MD, United States

**Keywords:** adeno-associated virus vector, deamidation, post-translational modification, crossreactivity, immune monitoring

## Abstract

During gene therapy trials, immune responses against adeno-associated virus (AAV) vectors are monitored by antibody assays that detect the humoral and T-cell mediated cellular responses to AAV vectors. T cell assays commonly utilize the collection of patients’ peripheral blood mononuclear cells (PBMCs) and stimulation with AAV-derived overlapping peptides. We recently described that spontaneous deamidation coincides with T cell epitopes in AAV capsids and that spontaneous deamidation may enhance or decrease immunogenicity in some individuals. This raised the concern for false negative results of antibody detection and PBMC immune monitoring assays because these assays use wild-type (WT) AAV or WT peptides for T cell re-stimulation and these peptides may not re-activate T cells that were stimulated with deamidated AAV capsid. To investigate this concern, we modeled the scenario by expanding T cells with deamidated peptides and evaluated the cross-reactivity of expanded T cells to WT peptides. In the majority of samples, cells that were expanded with deamidated peptides and restimulated with WT peptide had significantly lowered IL-2 and IFN-γ production. Spiking the four deamidated peptides to the WT peptide pool used for re-stimulation, restored the signal and corrected the performance of the assay. We also evaluated the impact of deamidation on anti AAV binding antibodies and did not observe a major impact on seroprevalence detection of AAV9. These data indicate that a high level of deamidation in AAV therapy may result in underestimation or even failure to detect immune responses against WT peptides during cellular immune monitoring.

## Introduction

The use of adeno-associated virus (AAV) vectors is growing rapidly among various fields including cancer, genetic disorders, *in vivo* gene editing and infectious diseases. However, concerns over the immunogenicity of the AAV capsid in humans were raised in clinical trials and impeded the therapeutic success of FDA regulated products (1, 2). Specifically, AAV vectors have been shown to engage both the innate and adaptive arms of the immune system with direct and indirect impact on the safety and efficacy of the gene therapy (3–5). Clinical data collected from multiple gene therapy trials revealed that T cells that are specific to epitopes on the AAV sequence, may activate and become cytotoxic towards AAV transduced cells (1, 6, 7). Such activity can result in liver toxicity, compromised efficacy and in some cases necessitate co-therapy of immune suppression regimen such as corticosteroids (8).

As a result, there is a need for a comprehensive characterization of the immune response against AAV vectors in gene therapy trials. The majority of conventional characterization focuses on adaptive immune monitoring which includes humoral and cellular characterization of the specific immune response towards the AAV vector before and after therapy (9, 10).

Cellular assays commonly utilize collection of patients derived PBMC at different time points before and after therapy, stimulation of these cells with large pools of AAV-derived overlapping peptides, and detection of T cell activation markers such as cytokine secretion (11). For immunogenicity monitoring of the humoral response, ELISA that detects binding antibodies (12) and various formats of cellular assays that detect neutralizing antibodies are commonly used (13).

Asparagine deamidation is one of the most common spontaneous post-translational modifications (PTM) (14), and it can cause the alteration of MHC binding or T cell receptor recognition, both of which have an important impact on immune recognition (15). In 2018, Giles et al. have demonstrated that recombinant AAV capsids undergo a high level of spontaneous deamidation in which the N+1 residue is glycine (i.e., NG pairs) (16). More recently, we performed *in silico* prediction as well as various immunological assays to study the effects of deamidation on the immunogenicity of AAV, and found that this deamidation may increase CD4 Th1 cell reactivity in some individuals or decrease in others (17). This raises the concern that during immune monitoring cellular assays, WT peptides that are used for restimulation would not cross-react with the T cells that were formed against the deamidated AAV capsid and may result in false negative results or decreased sensitivity of the assays. To investigate this concern, we analyzed the cross-reactivity of deamidated AAV capsid in T cell mediated ELISpot assays. To further investigate if AAV deamidation can impact humoral immune monitoring, we compared the binding titers of seropositive pre-existing serum samples by binding ELISAs to WT and to complete deamidated AAV capsids.

## Materials and methods

### Human PBMC samples

Peripheral mononuclear cells (PBMCs) were collected from apheresis samples of 16 healthy donors under protocols approved by the National Institutes of Health Institutional Review Board (CBER-047). Samples were isolated using gradient-density separation by Ficoll- Hypaque (GE Healthcare, Chicago, Illinois) and frozen in liquid nitrogen using RPMI media (Thermo Fisher Scientific) supplemented with 10% heat-inactivated human AB serum (MilliporeSigma) and 7.5% DMSO (Thermo Fisher Scientific).

### Peptide synthesis

All peptides were purified to >95% homogeneity by high-performance liquid chromatography (HPLC) (GenScript Biotech, New Jersey), and their composition and deamidation were confirmed twice by mass spectrometry, once during manufacture release and once after DMSO reconstitution and a freeze thaw cycle. Peptides were resuspended 0.05% DMSO and RPMI in a concentration of 20 µg/ml.

### *In vitro* expansion of PBMCs

PBMCs were thawed, resuspended in in RPMI media containing 5% heat-inactivated human serum, 1% Glutamax (Thermo Fisher Scientific), 1 mM sodium pyruvate (Thermo Fisher Scientific), 10 mM HEPES (Thermo Fisher Scientific), MEM Non-Essential Amino Acids (Thermo Fisher Scientific), and 1% penicillin/streptomycin (Thermo Fisher Scientific) at a concentration of 5×10^6^ cells/mL. Cells were stimulated with 5 μg/mL of WT or deamidated peptide pool and supplemented with 20 units of IL-2 (MilliporeSigma), 5 μg/mL of IL-7 (Biolegend, San Diego, CA), and 25 μg/mL of IL-15 (Biolegend) every 3-4 days for 10 days by gentle pipetting.

### ELISpot assay

After the *in vitro* expansion step, cells were harvested, washed, and plated in ELISpot plates that were pre-coated with anti-human IL-2 or IFN-γ antibodies (Mabtech, Cincinnati, OH). Cells were re-stimulated with 5 μg/mL of WT or deamidated peptides and incubated for 24 hours at 37°C. Negative controls were treated with medium, and positive controls were treated with phytohemagglutinin (PHA, MilliporeSigma). Secretions of IL-2 and IFN-γ following stimulation with peptides were detected using a secondary biotinylated anti-IL-2 or anti-IFN-γ antibody (Mabtech) followed by streptavidin, alkaline phosphatase conjugate (SA-ALP) (Mabtech), nitro blue tetrazolium and 5-bromo-4-chloro-3’-indolyl phosphate (BCIP/NBT) substrate (KPL, Thermo Fisher Scientific). We used the computer software (Immunospot 7.0; Cellular Technology Limited, Cleveland, OH) to measure the number of spots forming cells (SFC). The assay was performed in triplicate and repeated at least once for each donor.

### Sorting of activated CD4 T cells and monocytes

After the *in vitro* expansion step, cells were harvested, washed, and stained with Brilliant Violet (BV) 510-conjugated CD4 (clone SK3), fluorescein isothiocyanate (FITC)-conjugated CD3 (clone UCHT1), and R-phycoerythrin (R-PE)-conjugated CD154 (clone 24-31) for 20 minutes at 4°C. For monocyte isolation, freshly thawed PBMCs were stained with BV510-conjugated CD14 (clone M5E2). Cells were then washed twice and sorted using a BD FACSAria cell sorter by gating on CD3^+^CD4^+^CD154^+^ or CD14^+^ cells. Resulting cell fractions were washed and resuspended in 10% human serum containing media. In 96 well ELISpot plates, 25,000 of CD154^+^CD4^+^ T cells and 5000 of CD14^+^ cells were co-cultured with peptide stimulation, and ELISpot testing were performed as described above.

### Production of AAV vectors and quantification

AAV9 vectors were produced in free style 293F cells (Thermo Fisher Scientific, San Jose, CA) using a triple transfection method as previously described (17) and purified with iodixanol ultracentrifugation methods. AAV vector titration and purity was performed by real-time quantitative PCR (qPCR) and ELISA (PRAAV9, Progen, PA), followed by silver staining (Thermo Fisher Scientific). For qPCR, the following primers and probes were used: EGFP (Forward: 5/- AGCAAAGACCCCAACGAGAA-3/, Reverse: 5/- GGCGGCGGTCACGAA-3/and Probe: 5/- 6FAM-CGCGATCACATGGTCCTGCTGG-TAMRA-3/.

### Quantification of pre-existing AAV antibodies

Pre-existing anti-AAV9 antibodies in the healthy human serum were measured by standard ELISA. 96-well half area ELISA plates (Thermo Fisher) were coated overnight with WT or mutated (N57D, N329D, N452D, and N512D) AAV9 in boric acid buffer at a final concentration of 5 ×10^10^ vp/well. Plates were blocked with blocking buffer (PBS+3% bovine serum albumin) for 2 hours. Plates were then incubated with anti-human IgG antibodies conjugated with HRP (Jackson lab, Bar Harbor, ME) for 1 hour, followed by enzymatic development (3,3′,5,5′-tetramethylbenzidine (TMB)).

### Statistical analysis

Data are represented as the group mean ± SD. Statistical significance (α=0.05) was tested using GraphPad Prism, version 7.0 (GraphPad Software, San Diego, CA). Differences between groups were examined for statistical significance using ANOVA with Tukey’s multiple comparisons test.

## Results and discussion

### Stimulation with AAV9 peptides reveals rare cross-reactivity to deamidated peptides in PBMC stimulation

**Figure 1:**
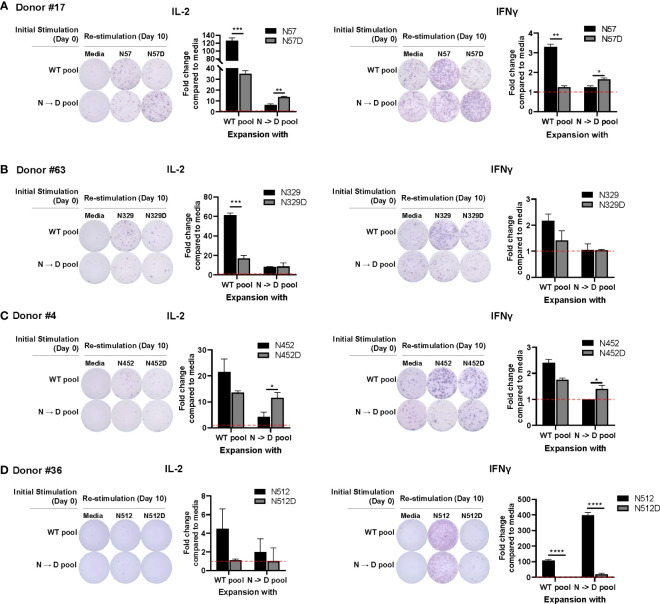
WT may not cross react with deamidated expanded peptides. Cells from healthy donors were expanded with either WT or deamidated peptides (N->D pool) for 10 days and re-stimulated with the reciprocal peptides (**A**; N57 or N57D, **B**; N329 or N329D, **C**; N452 or N452D, **D**; N512 or N512D). After 24 hours, IL-2 and IFNγ producing cells were detected by ELISpot assay. Red dash lines indicate the fold-change=1. Each bar show the mean ± SD. *p<0.05, **p<0.01, ***p<0.001, and ****p<0.0001. P values were determined by ANOVA with Tukey’s multiple comparisons test. Assay ran in duplicates and repeated at least once for every donor.

Prior studies have shown that recombinant AAV (rAAV) vectors undergo a high degree of spontaneous deamidation (16), and that this results in either reductions or enhancements in the immunogenicity of rAAV (17). This finding prompted us to investigate whether deamidated AAV cross-reacts with WT AAV. To investigate if T cells stimulated by deamidated peptides would cross-react and respond to WT peptides in an ELISpot assay, we enriched PBMCs from 16 healthy donors with WT or deamidated peptides and restimulated the cells with the reciprocal peptides. **Figure 1** shows 4 representative responses from donor #17 (**Figure 1A**), #63 (**Figure 1B**), #4 (**Figure 1C**), and #36 (**Figure 1D**) that responded to T cell epitopes in deamidation sites (N57, N329, N452, and N512). In donor #17, PBMCs that were expanded with WT or deamidated peptides and restimulated with the same peptides had strong IL-2 and IFN-γ responses. However, these responses were significantly decreased when the same cells were restimulated with reciprocal peptides (**Figure 1A**). In donor #63, PBMCs that were expanded with WT peptides and restimulated with deamidated peptides showed substantially lower IL-2 responses, whereas the opposite setting resulted in no differences (**Figure 1B**). In the other donor (#4, **Figure 1C**), there were significant differences when cells were expanded with deamidated peptides and restimulated with WT peptides. A different trend was observed in donor #36. PBMCs that were expanded with WT or deamidated peptides only responded to WT peptides, but not to deamidated peptides, regardless of the expansion (**Figure 1D**). This means that in cellular immune monitoring settings, donors #17 and #4 could have a false negative result to a deamidated AAV.

We further investigated the cross-reactivity of WT and deamidated peptides for other NG sites in other AAV serotypes. Deamidated peptides at N383 (from AAV1, 6, 8, 10) and N540 (from AAV8) showed low responses in IL-2 secretion in PBMC expanded with WT peptides, whereas WT peptides resulted in strong IL-2 production. There were no differences or responses in the opposite setting (data not shown).

**Figure 2:**
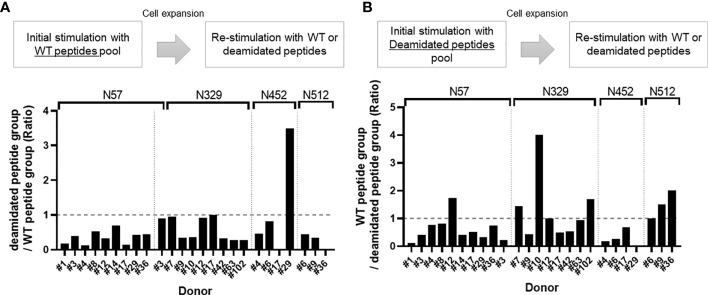
Cross-reactivity ratio between WT and deamidated peptides. Cells from healthy donors were stimulated with either WT **(A)** or deamidated **(B)** peptides pool and expanded for 10 days. Cells were re-stimulated with individual WT or deamidated peptides. After 24 hours, IL-2 producing cells were detected by ELISpot assay. **(A)** Y-axis indicates ratio of IL-2 producing cell in deamidated peptide-treated group to WT peptide-treated group. **(B)** Y-axis indicates ration of IL-2 producing cell in WT peptide-treated group to deamidated peptide-treated group. If the ratio is greater than 1, it was considered to have cross-reactivity.

To compare the responses of multiple donors, we calculated a cross-reactivity ratio (IL-2 spots after cognate stimulation/IL-2 secretion after reciprocal stimulation). A cross-reactivity ratio close to 1 indicates that WT peptides can successfully detect cellular response to WT or deamidated AAV. A cross-reactivity ratio that are close to 0 indicates that WT peptides do not cross react with deamidated peptides and that immune monitoring could miss a response to this peptide. **Figure 2** and **Table 1** show the cross-reactivity ratio for 16 samples. We observed low cross reactivity (<0.5) in the majority of the samples which indicates that a high level of deamidation of AAV therapy may result in underestimation or even failure to detect immune responses against these peptides during cellular immune monitoring.

**Table 1:** Cross-reactivity between WT and Deamidated peptide in immune responses.

Donor ID	1st stimulation: WT pool		1st stimulation: N→D pool	
	Ratio of N57D group to N57 group		Ratio of N57 group to N57D group	
	IL-2	IFN-γ	IL-2	IFN-γ
#1	0.17	1.05	0.11	1.07
#3	0.39	0.64	0.40	0.82
#4	0.13	0.76	0.77	0.70
#8	0.52	0.55	0.80	0.67
#12	0.33	0.79	1.73	1.08
#14	0.70	0.88	0.40	0.95
#17	0.14	0.29	0.51	0.77
#29	0.43	0.54	0.32	0.54
#36	0.44	1.25	0.75	0.03

Donor ID	1st stimulation: WT pool		1st stimulation: N→D pool	
	Ratio of N329D group to N329 group		Ratio of N329 group to N329D group	
	IL-2	IFN-γ	IL-2	IFN-γ
#3	0.90	0.98	0.21	0.50
#7	0.95	0.88	1.43	1.32
#9	0.35	1.01	0.44	1.03
#10	0.37	0.98	4.00	1.44
#12	0.91	1.84	1.00	0.44
#17	1.00	1.37	0.50	0.82
#42	0.32	0.92	0.54	0.88
#63	0.27	0.65	0.94	1.01
#102	0.28	0.92	1.70	0.94

Donor ID	1st stimulation: WT pool		1st stimulation: N→D pool	
	Ratio of N452D group to N452 group		Ratio of N452 group to N452D group	
	IL-2	IFN-γ	IL-2	IFN-γ
#4	0.47	0.54	0.18	0.41
#6	0.81	0.78	0.25	0.68
#17	0.03	0.47	0.69	1.05
#29	10.50	1.00	0.00	0.16

Donor ID	1st stimulation: WT pool		1st stimulation: N→D pool	
	Ratio of N512D group to N512 group		Ratio of N512 group to N512D group	
	IL-2	IFN-γ	IL-2	IFN-γ
#6	0.44	0.11	1.00	0.92
#9	0.35	0.06	1.50	4.14
#36	0.00	0.01	2.00	21.34

### Activated CD4 T cells by deamidated peptide cannot recognize WT peptide in some donors

**Figure 3:**
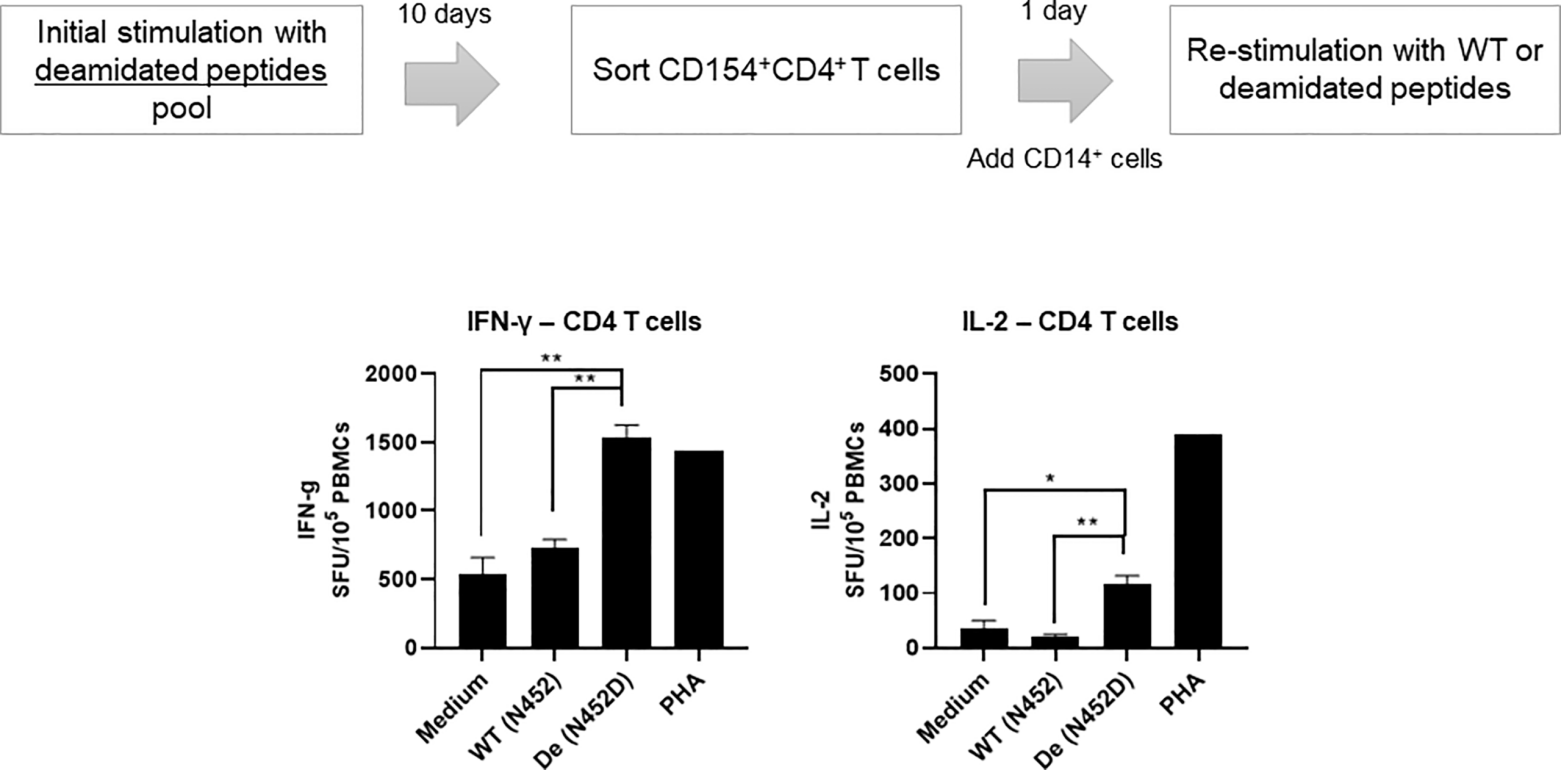
Activated CD4 T cells by deamidated peptide cannot recognize WT peptide. Cells from healthy donors (#6) were stimulated with deamidated peptides pool. After 10 days, sorted CD154^+^CD4^+^ T cells were re-stimulated with WT (N452) or deamidated (N452D) peptides in the presence of CD14^+^ cells (5:1). IL-2 and IFN-γ producing cells were detected by ELISpot assay. Each bar show the mean ± SD. *p<0.05, and **p<0.01. P values were determined by ANOVA with Tukey’s multiple comparisons test.

We have reported that deamidation sites (NG) contain CD4 T cell epitopes (17). To determine whether AAV reactive CD4 T cell that were activated by deamidated peptides react with WT peptides, we isolated activated CD154^+^CD4^+^ T cells after stimulation with deamidated peptides and re-stimulated them with WT peptides or deamidated peptides. **Figure 3** shows a representative response from donor #6. In this example, isolated CD154^+^CD4^+^ cells that were expanded with deamidated peptides and then restimulated with the same deamidated peptide showed 2.9-fold and 3.2-fold more spots than media control in IFN-γ and IL-2 ELISpot assays, respectively. Restimulating these cells with reciprocal WT peptides, on the other hand, did not show any significant responses, further indicating that WT peptides could fail to detect CD4 T cell response activated by deamidated epitopes in the capsid.

### Immune responses to deamidated AAV could be detected using a mixture of WT and deamidated peptides

**Figure 4:**
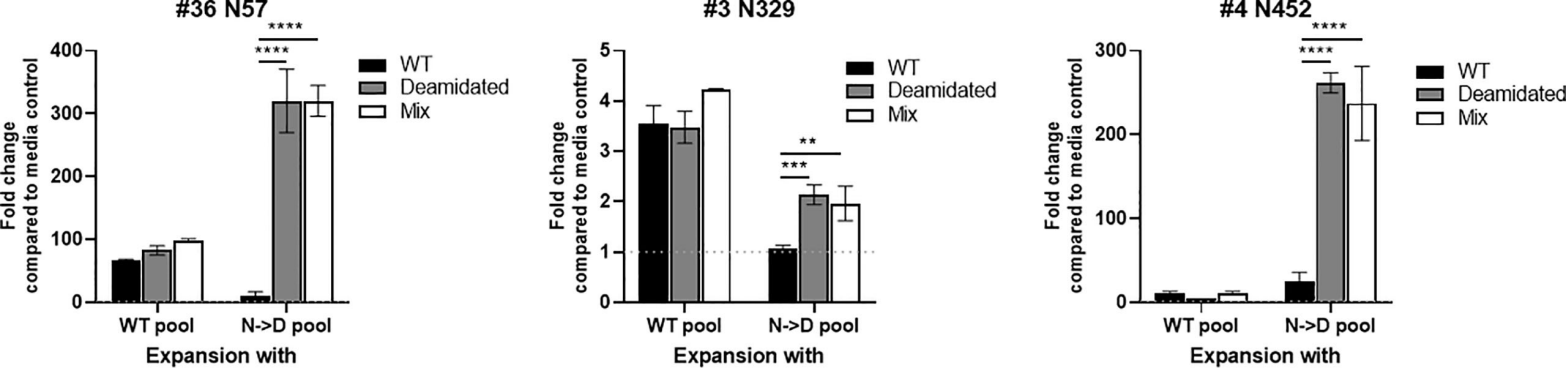
The immunogenic cells that are expended with deamidated pool can be detected using a mixture of WT and deamidated peptides. Cells from healthy donors were stimulated with either WT or deamidated (N->D) peptides pool and expanded for 10 days. Cells were re-stimulated with individual WT, deamidated or mixture of WT and deamidated peptides. After 24 hours, IFN-γ producing cells were detected by ELISpot assay. Y-axis indicates fold change compared to media control. Each bar show the mean ± SD. **p<0.01, ***p<0.01 and ****p<0.0001. P values were determined by ANOVA with Tukey’s multiple comparisons test.

We hypothesize that the risk of a false negative response in cellular immune monitoring assays due to the low cross reactivity between WT and deamidated peptides can be mitigated by spiking the few deamidated peptides to the mixture of WT peptides. To test this hypothesis, we compared the cellular response to WT and deamidated peptides to a mixture of both WT and deamidated peptides in three, three, and five donors for each of the NG sites, N57, N329, and N452, respectively that previously demonstrated a low cross-reactivity ratio of <0.5 (**Figure 2**). **Figure 4** shows representative responses of three donor samples after stimulation with peptides N57, N329, and N452. In most of the donors, enriched PBMCs with deamidated peptide pools responded to a peptide mixture of WT and deamidated peptides at a level comparable to the deamidated peptide alone despite the fact that the response to WT peptides in these cells was significantly lower. These results suggest that including deamidated peptides in the peptide master pools used in immune monitoring assays would improve the assay’s sensitivity. Of note, there were a few donors with comparable cross-reactivity between WT and deamidated peptides (data not shown). This fact can be explained by donor-to-donor variation which can be attributed to different HLA background of some donors or prior exposure of these donors to WT AAV9 virus.

### Pre-existing antibodies to AAV9 bind to deamidated AAV

**Figure 5:**
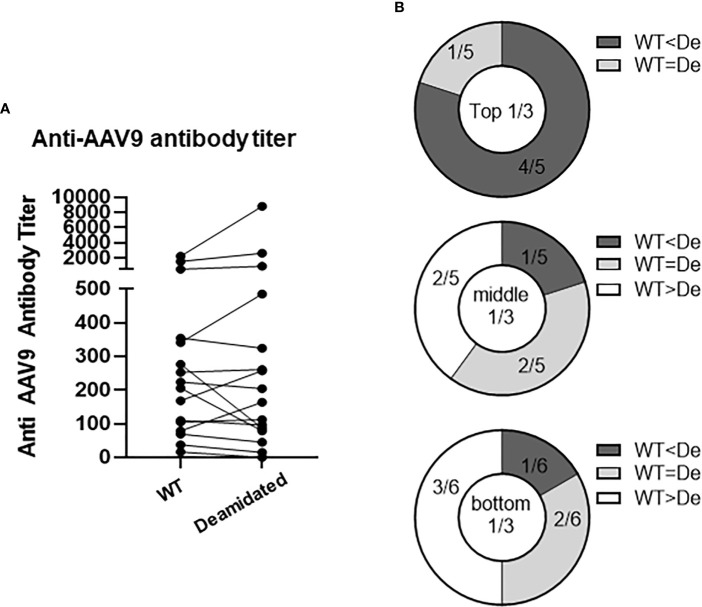
The titers of pre-existing antibodies are not affected by deamidation. Anti-AAV9 antibodies were detected in 16 human serum samples. WT or deamidated AAV9 capsids were coated passively onto a plate, serum samples were then added after blocking, and pre-existing antibodies were detected using a colorimetric reaction involving anti-human FC-HRP antibodies and TMB substrate. A titer was calculated by subtracting the signal from the sham ELISA and then interpolating the point where there is no longer change in OD signal in the reciprocal dilutions. **(A)** Anti-AAV titer against WT or deamidated (mutated) capsids in 16 healthy individuals. **(B)** Data were divided into 3 groups based on titer against WT capsids, then were compared to titer against mutated capsids. WT<De indicates that the titer against deamidated (mutated) capsids is more than 20% higher than the titer against WT capsids. WT<De indicates that the titer against deamidated (mutated) capsids is more than 20% lower than titer against WT capsids.

Testing pre-existing antibodies prior to gene transfer therapy is highly recommended due to the potential of antibody-mediated toxicity following AAV gene transfer. To investigate whether deamidated AAV could affect the sensitivity of detecting pre-existing anti-AAV9 antibodies, we compared the anti-AAV9 Ab titers in a binding ELISA assay using WT AAV9 and deamidated AAV9. To make 100% deamidated AAV9, four NG sites were mutated to DG (deamidated AAV9) (**Supplementary Figure 1**). The amino acid mutations in the four NG sites dramatically decreased the yield of the recombinant deamidated AAV9 vectors compared to WT AAV9 capsid (**Supplementary Figure 1**), as well as increased the ratio of empty and full AAV particles (**Supplementary Table 1**, Caspid/viral genome ratio) and its potency (**Supplementary Figure 2**). Freshly made WT AAV9 and deamidated AAV9 were coated onto a plate within 5-7 days of production, serum samples from 16 healthy donors were then added and serially diluted to detect the pre-existing antibodies against AAV9. **Figure 5A** shows that most serum samples had pre-existing antibodies to AAV9 but there were no significant differences in the antibody titers between two assays using WT and deamidated AAV9 for most of the samples. Notably, if pre-existing Ab titers against WT AAV9 were high, its titer against mutant AAV were much higher (**Figure 5B**), implying several hypotheses: 1) natural AAV9 may be spontaneously deamidated, and 2) high anti-AAV9 titer against deamidated AAV was a result of antibody cross reactivity to other AAV serotypes that already have DG amino acid sequence such as AAV1, AAV2, AAV3, AAV6, AAV7, AAV8 or AAV13 (**Supplementary Figure 3**).

## Concluding remarks

In this study, we demonstrate that the spontaneous deamidation-induced change from asparagine to aspartic acid in AAV capsid can compromise the performance of cellular immune monitoring assays such as ELISpot assays. Such assays that utilize peptide libraries spanning the sequence of WT AAV vectors can underestimate the cellular T cell responses to epitopes in deamidated sites in the AAV vector. To mitigate the risk of this underestimation we propose to add peptides that represent the deamidated sites by including peptides with an aspartic acid instead of asparagine at NG sites of the AAV capsid. These peptides can be spiked into the peptide mixture that spans the sequence of WT AAV. We showed that this simple and inexpensive solution results in an improved estimation of the cellular immune response to AAV derived epitopes.

While this study demonstrates the low cross-reactivity of wild-type and deamidated AAV, there are some limitations. First, it is not clear whether the *in vitro* expansion represents *de-novo* or boost because the seroprevalence of the donors was not determined. Our previous data showed that cells that responded to the deamidated peptides were CD4^+^ and mostly effector memory (CCR7^-^CD45RA^-^). Therefore, it is likely that most of the PBMC samples came from donors that were exposed to the AAV natural virus at some point (17). If the *in-vitro* expansion indeed represents a boost, we cannot determine to which AAV serotype the donors were exposed. Since the different serotypes have high sequence homology in the four deamidation sites, we would expect a similar immunological memory to the peptides that were used in this study, even if the PBMC donors were exposed to a serotype other than AAV9. Of note, this work focused on the change from asparagine to aspartic acid, even though deamidation can result in a change to either aspartic acid or isoaspartic acid. We chose to focus only on the aspartic acids because our previous work has demonstrated that peptides with isoaspartic in the same site does not result in any T cell activation (17). Due to the high doses used in clinical settings, even if only a fraction of the AAV particles will be deamidated to aspartic acid, the risk for an immune response against the deamidated epitope remains.

## Data availability statement

The original contributions presented in the study are included in the article/**Supplementary Material**. Further inquiries can be directed to the corresponding author.

## Ethics statement

The studies involving human participants were reviewed and approved by the National Institutes of Health (NIH) Institutional Review Board (CBER-047). The patients/participants provided their written informed consent to participate in this study.

## Author contributions

SB and SW performed the experiment and analyzed the results. SB and RM designed the experiment and wrote the manuscript. RM supervised the entire project. All authors contributed to the article and approved the submitted version.
