# Supplementary material for: Low cross reactivity between wild type and deamidated AAV can lead to false negative results in immune monitoring T-cell assays
Source: Front Immunol. 2023 Jul 4;14:1211529. doi: 10.3389/fimmu.2023.1211529 (PMC10352612; doi:10.3389/fimmu.2023.1211529)
Supplement: Supplementary file 1 [file DataSheet_1.pdf]

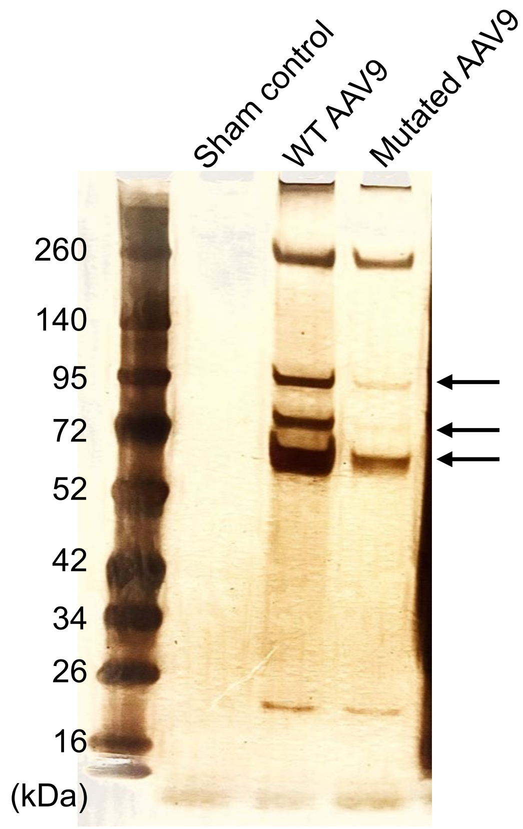

Supplementary Figure 1. Silver-stained SDS-polyacrylamide gel of final AAVs. Arrows indicate VP1, VP2, and VP3. Four site-point mutations (N57D/N329D/N452D/N512D) were introduced to make mutated AAV9. Sham control includes reagents that underwent all AAV manufacturing and purification steps but did not include any plasmids during the triple transfection step.

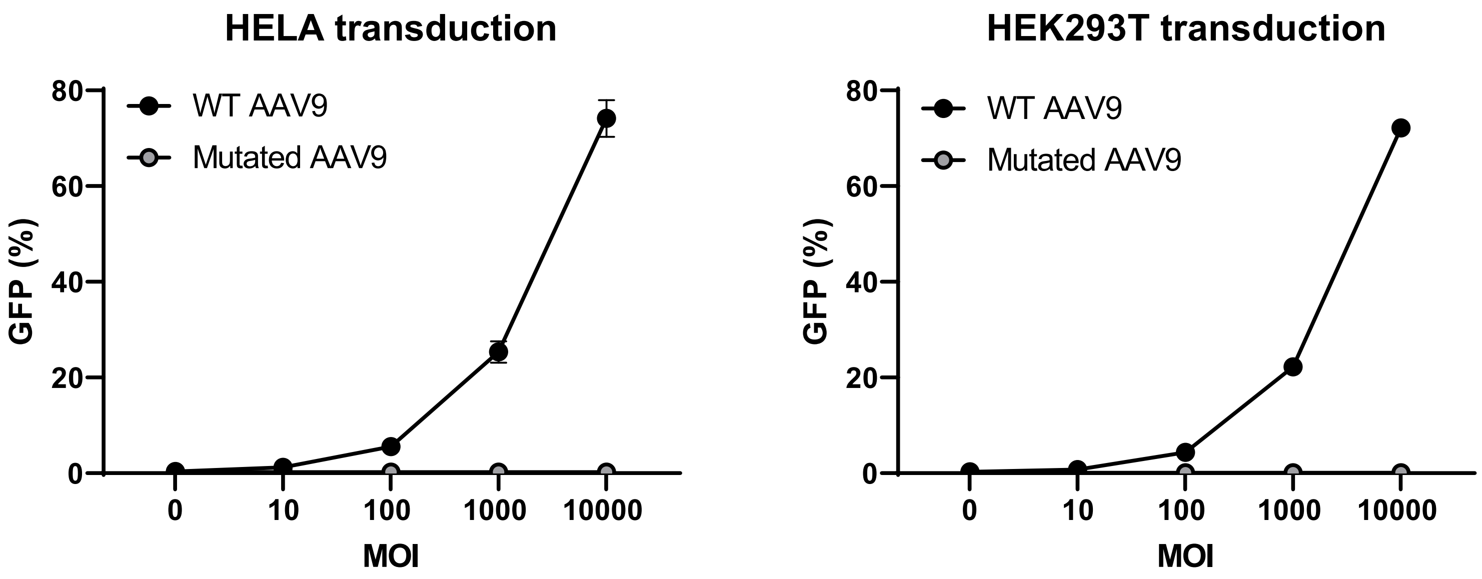

Supplementary Figure 2. Four aspartic acid mutations reduced the transduction efficacy. Freshly made WT AAV9 or mutated (N57D/N329D/N452D/N512D) AAV9 vectors at the MOIs indicated on the x-axis were incubated with HELA (A) or HEK293T (B) cells for 3 days, and the GFP expression was measured by flow cytometry.

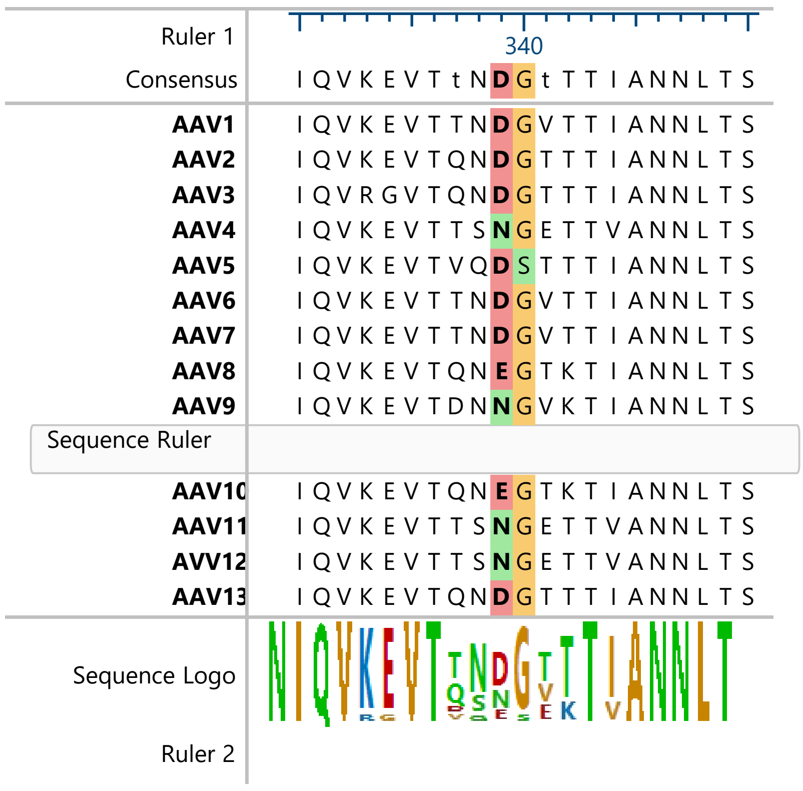

Supplementary Figure 3. Amino acid sequences of the NG site in other AAV serotypes. Capsid amino acids of AAV1-13 were aligned using DNASTAR program.

Supplementary Table 1. Quantification of final AAVs

AAV9	Quantification method		Capsid/viral genome ratio
	qPCR	ELISA	
WT	$2.0 \times 10^{11} \pm 7.7 \times 10^{10}$	$1.9 \times 10^{12} \pm 8.2 \times 10^{11}$	1:9 ~ 1:10
Mutated (N57D/N329D/N452D/N512D)	$1.7 \times 10^{9} \pm 1.5 \times 10^{8}$	$2.2 \times 10^{11} \pm 4.2 \times 10^{10}$	1:116 ~1:143

* Both WT and mutated total yield was 3.5 mL.
